# Author Correction: Associations of semaglutide with incidence and recurrence of alcohol use disorder in real-world population

**DOI:** 10.1038/s41467-024-49655-6

**Published:** 2024-06-18

**Authors:** William Wang, Nora D. Volkow, Nathan A. Berger, Pamela B. Davis, David C. Kaelber, Rong Xu

**Affiliations:** 1 Center for Science, Health, and Society, Case Western Reserve University School of Medicine, Cleveland, OH USA; 2 National Institute on Drug Abuse, National Institutes of Health, Bethesda, MD USA; 3 Center for Community Health Integration, Case Western Reserve University School of Medicine, Cleveland, OH USA; 4 Center for Clinical Informatics Research and Education, The MetroHealth System, Cleveland, OH USA; 5 Center for Artificial Intelligence in Drug Discovery, Case Western Reserve University School of Medicine, Cleveland, OH USA

**Keywords:** Addiction, Therapeutics, Clinical pharmacology, Drug safety

Correction to: *Nature Communications* 10.1038/s41467-024-48780-6, published online 28 May 2024

In this article in the abstract the word clinical was misspelt as clinicl. Additionally, on page 2 of the article the sentence ‘Compared to naltrexone or topiramate, semaglutide was associated with a significantly lower risk of incident AUD diagnosis’ was incorrect and should have been ‘Compared to naltrexone or topiramate, semaglutide was associated with a significantly lower risk of recurrent AUD diagnosis’. Finally, on page 8 the sentence ‘Fourth the follow-up time for the main analyses was 8 months’ should have read ‘Fourth the follow-up time for the main analyses was 12 months.’ The original article has been corrected.

