# Supplementary material for: Associations of semaglutide with incidence and recurrence of alcohol use disorder in real-world population
Source: Nat Commun. 2024 May 28;15:4548. doi: 10.1038/s41467-024-48780-6 (PMC11133479; doi:10.1038/s41467-024-48780-6)
Supplement: Supplementary file 1 — Supplementary Information [file 41467_2024_48780_MOESM1_ESM.pdf]

Supplementary Table 1: Characteristics of the study population with T2DM and no prior history of AUD. SMD – standardized mean differences. *SMD less than 0.1, indicating cohort balance.

	**Before Propensity-Score Matching**			**After Propensity-Score Matching**		
	**semaglutide cohort**	**non-GLP-1RA anti-diabetes medications cohort**	**SMD**	**semaglutide cohort**	**non-GLP-1RA anti-diabetes medications cohort**	**SMD**
**Total number**	25,686	573,117		25,670	25,670	
**Age at index event (years, mean±SD)**	58.2 ± 11.9	63.1± 13.3	0.39 *	58.2 ± 11.9	57.7 ± 13.6	0.04
**Sex (%)**						
Female	45.8	44.7	0.02	45.8	44.7	0.02
Male	46.1	50.1	0.08	46.1	47.0	0.02
Unknown	8.1	5.2	0.03	8.1	8.3	0.006
**Ethnicity (%)**						
Hispanic/Latinx	6.6	9.1	0.09	6.6	6.3	0.01
Not Hispanic/Latinx	63.3	56.3	0.14 *	63.3	63.9	0.01
Unknown	30.0	34.6	0.09	30.0	29.7	0.007
**Race (%)**						
Asian	4.7	5.5	0.04	4.7	4.7	0.002
Black	14.7	17.1	0.06	14.7	14.6	0.005
White	60.2	57.7	0.05	60.2	60.3	0.001
Unknown	15.2	14.9	0.007	15.2	15.2	0.002
**Marital status (%)**						
Never Married	10.4	10.4	0.003	10.4	10.3	0.005
Divorced	5.3	4.5	0.04	5.3	5.2	0.003
Widowed	4.9	6.1	0.06	4.9	4.7	0.01
**Adverse socioeconomic determinants of health (%)**	2.9	2.1	0.05	2.9	2.8	0.004
**Problems related to lifestyle (%)**	6.7	4.1	0.11 *	6.6	6.7	0.001
**Family history of mental and behavioral disorders**	0.4	0.3	0.01	0.4	0.4	0.004
**Pre-existing medical conditions, procedures, medications (%)**						
Depression	21.5	13.2	0.22 *	21.5	20.7	0.02
Mood disorders	24.9	16.1	0.22 *	24.9	24.1	0.02
Anxiety disorders	26.1	15.8	0.26 *	26.1	25.3	0.02
Psychotic disorders	1.3	2.0	0.06	1.3	1.2	0.01
Behavioral disorders	5.4	1.8	0.20 *	5.3	5.1	0.01
Disorders of adult personality and behavior	0.9	0.5	0.05	0.9	0.9	<.001
Behavioral and emotional disorders with onset in	1.9	0.8	0.09	1.9	1.8	0.01

childhood and adolescence						
Conduct disorders	0.1	0.1	0.008	0.1	0.2	0.01
Symptoms and signs involving emotional state	3.3	2.0	0.08	3.3	3.3	0.003
Chronic pain	20.8	10.7	0.28*	20.8	19.8	0.03
Cancer	32.1	18.7	0.31*	32.1	31.0	0.02
Cannabis use disorder	0.8	1.1	0.04	0.8	0.8	<.001
Opioid use disorder	1.2	1.0	0.02	1.2	1.2	<.001
Tobacco use disorder	13.2	13.5	0.009	13.2	13.1	0.003
Cocaine use disorder	0.3	0.6	0.03	0.3	0.4	0.002
Other stimulant disorders	0.3	0.5	0.02	0.3	0.3	0.005
Other psychoactive substance related disorders	0.8	0.7	0.005	0.8	0.8	0.001
Hypertension	86.0	87.1	0.03	86.0	85.1	0.02
Disorders of lipoprotein	85.9	65.8	0.49*	85.9	86.1	0.004
Hyperlipidemia	64.7	50.1	0.30*	64.7	64.1	0.01
Hypercholesterolemia	27.0	16.6	0.25*	27.0	26.2	0.02
Ischemic heart diseases	23.7	26.8	0.07	23.7	22.6	0.03
Other forms of heart disease	30.3	33.5	0.07	30.3	28.7	0.03
Cerebral infarction	4.6	5.8	0.06	4.6	4.3	0.01
Cerebrovascular diseases	9.9	11.6	0.06	9.9	9.3	0.02
Morbid obesity	28.1	12.0	0.41*	28.1	27.5	0.01
Obesity, unspecified	39.9	18.8	0.48*	39.8	38.9	0.02
Other obesity	7.7	2.0	0.27*	7.7	7.2	0.02
Substance abuse treatment	0.0	0.0	0.003	0.0	0.0	<.001
Psychotherapy	2.1	0.6	0.13*	2.1	2.1	0.002
Baclonfen	3.1	1.5	0.10*	3.1	2.8	0.02
Gabapentin	23.1	14.0	0.24*	23.0	22.2	0.02
Naltrexone	0.6	0.1	0.09	0.6	0.6	0.003
Topiramate	3.4	1.0	0.16*	3.4	3.1	0.01
**Medical visit types (%)**						
Outpatient	88.7	63.9	0.61*	88.7	89.5	0.03
Inpatient	31.5	36.6	0.11*	31.5	29.8	0.04
Emergency	37.0	27.7	0.20*	36.9	35.6	0.03

Virtual	4.6	1.6	0.17*	4.6	4.4	0.01

Supplementary Table 2: Characteristics of the study population with T2DM and a prior history of AUD. SMD – standardized mean differences. *SMD less than 0.1, indicating cohort balance.

	**Before Propensity-Score Matching**			**After Propensity-Score Matching**		
	**semaglutide cohort**	**non-GLP-1RA anti-diabetes medications cohort**	**SMD**	**semaglutide cohort**	**non-GLP-1RA anti-diabetes medications cohort**	**SMD**
**Total number**	668	21,445		653	653	
**Age at index event (mean±SD)**	57.1 ± 11.0	57.2± 11.5	0.008	57.2 ± 11.1	57.6 ± 11.5	0.03
**Sex (%)**						
Female	26.2	19.9	0.15*	25.6	27.1	0.04
Male	68.0	76.0	0.18*	68.6	68.0	0.01
Unknown	5.8	4.1	0.08	5.8	4.9	0.04
**Ethnicity (%)**						
Hispanic/Latinx	7.0	8.8	0.07	7.0	10.0	0.10*
Not Hispanic/Latinx	66.8	60.4	0.13*	66.9	65.5	0.03
Unknown	26.2	30.8	0.10*	26.0	24.5	0.03
**Race (%)**						
Asian	4.5	2.5	0.11*	4.6	5.5	0.04
Black	16.3	20.1	0.10*	16.4	17.9	0.04
White	57.0	57.8	0.02	57.0	53.9	0.06
Unknown	16.3	14.4	0.05	16.2	15.9	0.008
**Marital status (%)**						
Never Married	11.7	16.1	0.13*	11.8	11.9	0.005
Divorced	8.1	6.4	0.06	7.7	6.3	0.05
Widowed	3.1	3.1	0.001	3.2	4.0	0.04
**Adverse socioeconomic determinants of health (%)**	12.7	9.8	0.09	12.3	14.1	0.05
**Problems related to lifestyle (%)**	24.4	16.4	0.20*	24.2	26.2	0.05
**Family history of mental and behavioral disorders**	3.1	1.6	0.10*	2.9	3.4	0.03
**Pre-existing medical conditions, procedures, medications (%)**						
Depression	47.9	28.5	0.41*	47.0	48.1	0.02
Mood disorders	55.2	35.8	0.40*	54.5	56.5	0.04
Anxiety disorders	52.1	32.7	0.40*	51.1	51.0	0.003
Psychotic disorders	7.5	7.4	0.002	7.2	8.0	0.03

Behavioral disorders	14.7	3.4	0.40 *	13.8	14.7	0.03
Disorders of adult personality and behavior	5.5	3.0	0.12 *	5.2	5.5	0.01
Behavioral and emotional disorders with onset in childhood and adolescence	6.0	2.4	0.18 *	5.8	5.8	<.001
Conduct disorders	1.5	0.7	0.08	1.5	1.5	<.001
Symptoms and signs involving emotional state	16.8	12.0	0.14 *	15.9	15.8	0.004
Chronic pain	40.7	18.8	0.49 *	39.7	41.0	0.03
Cancer	48.2	20.4	0.61 *	47.3	47.8	0.009
Cannabis use disorder	9.1	10.1	0.03	9.2	10.3	0.04
Opioid use disorder	8.7	6.7	0.07	8.3	10.0	0.06
Tobacco use disorder	45.4	47.1	0.04	45.3	47.0	0.03
Cocaine use disorder	8.8	10.0	0.04	8.6	10.9	0.08
Other stimulant disorders	3.6	4.2	0.03	3.7	3.5	0.008
Other psychoactive substance related disorders	13.8	10.1	0.11 *	13.0	12.9	0.005
Hypertension	93.7	88.8	0.18 *	93.7	93.9	0.006
Disorders of lipoprotein	88.5	58.2	0.73 *	88.2	87.4	0.02
Hyperlipidemia	77.4	48.2	0.63 *	76.9	76.4	0.01
Hypercholesterolemia	36.4	15.6	0.49 *	35.4	33.8	0.03
Ischemic heart diseases	36.8	27.6	0.20 *	36.4	35.2	0.03
Other forms of heart disease	51.3	41.3	0.20 *	50.8	49.9	0.02
Cerebral infarction	9.7	7.7	0.07	9.5	9.3	0.005
Cerebrovascular diseases	20.7	15.6	0.13 *	20.2	19.1	0.03
Morbid obesity	34.0	11.4	0.56 *	32.9	29.9	0.07
Obesity, unspecified	58.8	23.2	0.78 *	57.9	58.8	0.02
Other obesity	10.2	2.1	0.34 *	9.5	11.2	0.06
Substance abuse treatment	4.8	5.5	0.03	4.4	4.3	0.007
Psychotherapy	14.5	3.2	0.41 *	13.3	13.3	<.001
Acamproate	2.4	0.7	0.14 *	2.1	1.8	0.02
Disulfiram	1.8	0.5	0.12 *	1.5	1.8	0.02
Baclonfen	7.5	2.7	0.22 *	6.9	8.6	0.06
Gabapentin	38.2	21.0	0.38 *	37.4	39.8	0.05

Naltrexone	5.1	2.0	0.17*	4.6	4.7	0.007
Topiramate	6.4	1.6	0.25*	6.1	6.3	0.006
**Medical visit types (%)**						
Outpatient	94.0	63.5	0.80*	93.9	94.3	0.02
Inpatient	63.3	58.5	0.10*	62.8	61.1	0.04
Emergency	65.7	44.9	0.43*	64.9	66.3	0.03
Virtual	7.6	3.5	0.18*	7.7	6.1	0.06

Supplementary Table 3: Clinical diagnosis, and other codes used in TriNetX platform that are used to determine the status of variables for study population definitions, exposures, outcomes, and for propensity-score matching for cohorts.

**Eligibility (inclusion and exclusion criteria)**	AUD	• Alcohol use disorders (ICD-10 code: F10)	present/absent
	Obesity	• Obesity due to excess calories (ICD-10 code: E66.0)<br>• Morbid (severe) obesity due to excess calories (ICD-10 code: E66.01)<br>• Drug-induced obesity (ICD-10 code: E66.1)<br>• Morbid (severe) obesity with alveolar hypoventilation (ICD-10 code: E66.2)<br>• Other obesity (ICD-10 code: E66.8)<br>• Obesity, unspecified (ICD-10 code: E66.9)<br>• Body mass index [BMI] 30-39, adult (ICD-10 code: Z68.3)<br>• Body mass index [BMI] 40 or greater, adult (ICD-10 code: Z68.4)<br>• Body mass index [BMI] 30.0-30.9, adult (ICD-10 code: Z68.30)<br>• Body mass index [BMI] 31.0-31.9, adult (ICD-10 code: Z68.31)<br>• Body mass index [BMI] 32.0-32.9, adult (ICD-10 code: Z68.32)<br>• Body mass index [BMI] 33.0-33.9, adult (ICD-10 code: Z68.33)<br>• Body mass index [BMI] 34.0-34.9, adult (ICD-10 code: Z68.34)<br>• Body mass index [BMI] 35.0-35.9, adult (ICD-10 code: Z68.35)<br>• Body mass index [BMI] 36.0-36.9, adult (ICD-10 code: Z68.36)<br>• Body mass index [BMI] 37.0-37.9, adult (ICD-10 code: Z68.37)<br>• Body mass index [BMI] 38.0-38.9, adult (ICD-10 code: Z68.38)<br>• Body mass index [BMI] 39.0-39.9, adult (ICD-10 code: Z68.39)<br>• Body mass index [BMI] 40.0-44.9, adult (ICD-10 code: Z68.41)<br>• Body mass index [BMI] 45.0-49.9, adult (ICD-10 code: Z68.42)	present/absent

		• Body mass index [BMI] 50.0-59.9, adult (ICD-10 code: Z68.43)<br>• Body mass index [BMI] 60.0-69.9, adult (ICD-10 code: Z68.44)<br>• Body mass index [BMI] 70 or greater, adult (ICD-10 code: Z68.45)	
	T2DM	Type 2 diabetes mellitus (ICD-10 code: E11)	present/absent
	Obesity-related comborbidities	• Type 2 diabetes mellitus (ICD-10 code: E11)<br>• Hypertensive diseases (ICD-10 code: E11)<br>• Disorders of lipoprotein metabolism and other lipidemias (ICD-10 code: E78)<br>• Pure hypercholesterolemia (ICD-10 code: E78.0)<br>• Mixed hyperlipidemia (ICD-10 code: E78.2)<br>• Other hyperlipidemia (ICD-10 code: E78.2)<br>• Hyperlipidemia, unspecified (ICD-10 code: E78.5)<br>• Ischemic heart diseases (ICD-10 code: I20-I25)<br>• Other forms of heart disease (ICD-10 code: I30-I5A)<br>• Cerebral infarction (ICD-10 code: I63) Cerebrovascular diseases (ICD-10 code: I60-I69)	present/absent
	Bariatric surgery	• Bariatric surgery status (ICD-10 code: Z98.84)<br>• Gastrointestinal System / Bypass / Stomach (ICD10 code: :0D16	present/absent
	Other GLP-1RA medications	• lixisenatide (RxNorm code: 1440051)<br>• albiglutide (RxNorm code: 1534763)<br>• dulaglutide (RxNorm code: 1551291)<br>• liraglutide (RxNorm code: 475968)<br>• exenatide (RxNorm code: 60548)<br>• tirzepatide (RxNorm code: 2601723)	present/absent
**Treatment strategies**	Semaglutide	Semaglutide (RxNorm code: 1991302)	present/absent
	non-GLP-1RA anti-obesity medications	• Orlistat (RxNorm code: 37925)<br>• Phentermine: RxNorm code 8152<br>• Bupropion: RxNorm code 42347<br>• Naltrexone: RxNorm code 7243<br>• Topiramate: RxNorm code 38404	present/absent
	Naltrexone/Topiramate	• Naltrexone: RxNorm code 7243<br>• Topiramate: RxNorm code 38404	
	Non- GLP-1RA anti-diabetes medications	• Drugs used in diabetes (ATC code: A10) with GLP-1RA medications (lixisenatide, albiglutide, dulaglutide, liraglutide, exenatide, tirzepatide) excluded.	present/absent
**Outcome**	AUD diagnosis	Alcohol use disorders (ICD-10 code: F10)	present/absent
**Demographics**<br><br>**(variables to be propensity-score matched)**	Age at the index event	Age	continuous
	Female	F	present/absent
	Male	M	present/absent
	Asian	Asian (Demographics: 2028-9)	present/absent
	Black or African American	Black or African American (Demographics: 2054-5)	present/absent

	White	White (Demographics: 2106-3)	present/absent
	Hispanic/Latino	Hispanic or Latino (Demographics: 2135-2)	present/absent
	Not Hispanic or Latino	Not Hispanic or Latino (Demographics: 2186-5)	present/absent
	Unknown race	Unknown Race (Demographics: 2131-1)	present/absent
	Unknown ethnicity	Unknown Ethnicity (Demographics: UN)	present/absent
	Divorced	Divorced (Demographics: D)	present/absent
	Widowed	Widowed (Demographics: W)	present/absent
	Never married	Never Married (Demographics: S)	present/absent
**Adverse socioeconomic determinants of health, lifestyle factors, medical conditions, medication prescriptions, (anytime to before or on the day of the index event)**	Adverse socioeconomic and psychosocial circumstances	Persons with potential health hazards related to socioeconomic and psychosocial circumstances (ICD-10 code: Z55-Z65)<br>• Problems related to education and literacy (ICD-10 code: Z55)<br>• Problems related to employment and unemployment (ICD-10 code: Z56)<br>• Problems related to housing and economic circumstances (ICD-10 code: Z59)<br>• Problems related to social environment (ICD-10 code: Z60)<br>• Problems related to upbringing (ICD-10 code: Z62)<br>• Other problems related to primary support group, including family circumstances (ICD-10 code: Z63)<br>• Problems related to certain psychosocial circumstances ICD-10 code: Z64)<br>• Problems related to other psychosocial circumstances (ICD-10 code: Z65)	present/absent
	Problems related to lifestyle	Problems related to lifestyle (ICD-10 code: Z72)<br>• Tobacco use (ICD-10 code: Z72.0)<br>• Lack of physical exercise ((ICD-10 code: Z72.3)<br>• Inappropriate diet and eating habits (ICD-10 code: Z72.4)<br>• High risk sexual behavior (ICD-10 code: Z72.5)<br>• Gambling and betting (ICD-10 code: Z72.6)<br>• Other problems related to lifestyle (ICD-10 code: Z72.8)<br>• Problem related to lifestyle, unspecified (ICD-10 code: Z72.9)	present/absent
	Depression	Depressive episode (ICD-10 code: F32)	present/absent
	Mood disorders	Mood [affective] disorders (ICD-10 code: F30-F39)	present/absent
	Anxiety, dissociative, , somatoform and other nonpsychotic mental disorders including PTSD	Anxiety, dissociative, stress-related, somatoform and other nonpsychotic mental disorders (ICD-10 code: F40-F48)	present/absent
	Schizophrenia, schizotypal, delusional, and other non-mood psychotic disorders	Schizophrenia, schizotypal, delusional, and other non-mood psychotic disorders (ICD-10 code: F20-F29)	present/absent

	Behavioral disorders including sleep disorders	Behavioral syndromes associated with physiological disturbances and physical factors (ICD-10 code: F50-F59)	present/absent
	Disorders of adult personality and behavior including impulse and gender identity disorders	Disorders of adult personality and behavior (ICD-10 code: F60-F69)	present/absent
	Behavioral and emotional disorders with onset usually occurring in childhood and adolescence	Behavioral and emotional disorders with onset usually occurring in childhood and adolescence (ICD-10 code: F90-F98)	present/absent
	Conduct disorders	Conduct disorders ((ICD-10 code: F90)	present/absent
	Symptoms and signs involving emotional state	Symptoms and signs involving emotional state (ICD-10 code: R45)	present/absent
	Chronic pain	Chronic pain, not elsewhere classified (ICD-10 code: G89.2)	present/absent
	Cannabis related disorders	Cannabis related disorders (ICD-10 code: F12)	
	Opioid use disorder	Opioid use disorders (ICD-10 code: F11)	present/absent
	Tobacco use disorder	Nicotine dependence (ICD-10 code: F17)	present/absent
	Cocaine use disorder	Cocaine use disorders (ICD-10 code: F14)	present/absent
	Other stimulant disorders	Other stimulant disorders (ICD-10 code: F15)	present/absent
	Other psychoactive substance related disorders	Other psychoactive substance related disorders (ICD-10 code: F19)	present/absent
	Family history of mental and behavioral disorders	Family history of mental and behavioral disorders (ICD-10 code: Z81)	present/absent
	Substance Abuse Treatment	Substance Abuse Treatment (HCPCS Code: H)	present/absent
	Psychotherapy Services and Procedures	Psychotherapy Services and Procedures (CPT code: 1021137)	present/absent
	Acamprosate	acamprosate (RxNorm code: 82819)	present/absent
	Disulfiram	disulfiram (RxNorm code: 3554)	present/absent
	Baclofen	baclofen (RxNorm code: 1292)	present/absent
	gabapentin	gabapentin (RxNorm code: 25480)	present/absent
	Outpatient visit	Visit: Ambulatory (visit type code: AMB)	present/absent
	Inpatient visit	Visit: Inpatient Encounter (visit type code: IMP)	present/absent
	Emergency visit	Visit: Emergency (visit type code: EMER)	present/absent
	Virtual visit	Visit: Virtual (visit type code: VR)	present/absent
