# Supplementary material for: Associations of semaglutide with incidence and recurrence of alcohol use disorder in real-world population
Source: Nat Commun. 2024 May 28;15:4548. doi: 10.1038/s41467-024-48780-6 (PMC11133479; doi:10.1038/s41467-024-48780-6)
Supplement: Supplementary file 3 — Reporting Summary [file 41467_2024_48780_MOESM3_ESM.pdf]

nature portfolio

Corresponding author(s): Rong Xu & Nora D Volkow

Last updated by author(s): Apr 4, 2024

Reporting Summary

Nature Portfolio wishes to improve the reproducibility of the work that we publish. This form provides structure for consistency and transparency in reporting. For further information on Nature Portfolio policies, see our Editorial Policies and the Editorial Policy Checklist.

Statistics

For all statistical analyses, confirm that the following items are present in the figure legend, table legend, main text, or Methods section.

n/a	Confirmed	
☐	☒	The exact sample size (*n*) for each experimental group/condition, given as a discrete number and unit of measurement
☒	☐	A statement on whether measurements were taken from distinct samples or whether the same sample was measured repeatedly
☐	☒	The statistical test(s) used AND whether they are one- or two-sided<br>*Only common tests should be described solely by name; describe more complex techniques in the Methods section.*
☐	☒	A description of all covariates tested
☒	☐	A description of any assumptions or corrections, such as tests of normality and adjustment for multiple comparisons
☐	☒	A full description of the statistical parameters including central tendency (e.g. means) or other basic estimates (e.g. regression coefficient) AND variation (e.g. standard deviation) or associated estimates of uncertainty (e.g. confidence intervals)
☐	☒	For null hypothesis testing, the test statistic (e.g. *F*, *t*, *r*) with confidence intervals, effect sizes, degrees of freedom and *P* value noted<br>*Give P values as exact values whenever suitable.*
☒	☐	For Bayesian analysis, information on the choice of priors and Markov chain Monte Carlo settings
☒	☐	For hierarchical and complex designs, identification of the appropriate level for tests and full reporting of outcomes
☒	☐	Estimates of effect sizes (e.g. Cohen's *d*, Pearson's *r*), indicating how they were calculated

Our web collection on statistics for biologists contains articles on many of the points above.

Software and code

Policy information about availability of computer code

Data collection

This study used population-level aggregate and HIPAA de-identified data collected by the TriNetX platform and available from TriNetX, LLC (https://trinetx.com/), but third-party restrictions apply to the availability of these data. The data were used under license for this study with restrictions that do not allow for the data to be redistributed or made publicly available. To gain access to the data, a request can be made to TriNetX (join@trinetx.com), but costs may be incurred, and a data-sharing agreement may be necessary. Data specific to this study including diagnosis codes and cohort characteristics in aggregated format are included in the manuscript as tables, figures, and supplementary files. Data through the TriNetX platform is queried in real-time with results being returned typically in seconds to minutes. Data from the underlying electronic health records of participating healthcare organizations is refreshed in the TriNetX platform from daily to every couple of months depending on the healthcare organization.

We used built-in statistical and informatics functions within the TriNetX Analytics Platform37 (Research US Collaborative Network) to analyze aggregated and de-identified patient electronic health records (EHRs). Analyses were performed on January 26, 2024. At the time of this study, TriNetX Research US Collaborative Network contained EHRs of 105.3 million patients from 61 healthcare organizations, most of which are large academic medical institutions, in the US across 50 states: 25%, 17%, 41%, and 12% in the Northeast, Midwest, South, West, respectively, and 5% unknown region.

TriNetX de-identifies and aggregates EHRs from contributing healthcare systems completes an intensive data preprocessing stage to minimize missing values, maps the data to a common clinical data model, and provides web-based analytics tools to analyze patient EHRs. All variables are either binary, categorical, or continuous but essentially guaranteed to exist. Missing sex, race, and ethnicity values are represented using “Unknown Sex”, “Unknown race” and “Unknown Ethnicity”, respectively. For other variables (e.g., medical conditions, medications, procedures, lab tests, and socio-economic determinant health), the value is either present or absent, and “missing” is not pertinent.

Data analysis

All the statistical analyses in this study including propensity-score matching, and Cox proportional hazards used web-baed built-in functions within the TriNetX Analytics Platform that are implemented using Survival 3.2-3 in R 4.0.2 and libraries/utilities for data science and statistics in Python 3.7 and Java 11.0.16. Data and code to recreate figures in the study can be accessed at https://github.com/bill-pipi/semaglutide_AUD

For manuscripts utilizing custom algorithms or software that are central to the research but not yet described in published literature, software must be made available to editors and reviewers. We strongly encourage code deposition in a community repository (e.g. GitHub). See the Nature Portfolio guidelines for submitting code & software for further information.

Data

Policy information about availability of data

All manuscripts must include a data availability statement. This statement should provide the following information, where applicable:
- Accession codes, unique identifiers, or web links for publicly available datasets
- A description of any restrictions on data availability
- For clinical datasets or third party data, please ensure that the statement adheres to our policy

We used built-in statistical and informatics functions within the TriNetX Analytics Platform
available from TriNetX, LLC (https://trinetx.com/). Third-party restrictions apply to the availability of these data, which do not allow for the EHR data to be redistributed or made publicly available. To gain access to the EHR data or the analytics platform to analyze the data, a request can be made to TriNetX (join@trinetx.com), but costs might be incurred, and a data-sharing agreement would be necessary. Data specific to this study including diagnosis codes, cohort characteristics in aggregated format as well as data and codes to recreate all the figures are included in the manuscript, supplementary files and at https://github.com/bill-pipi/semaglutide_AUD

Research involving human participants, their data, or biological material

Policy information about studies with human participants or human data. See also policy information about sex, gender (identity/presentation), and sexual orientation and race, ethnicity and racism.

Reporting on sex and gender

Self-reported sex (female, male) data in TriNetX comes from the underlying clinical EHR systems of the contributing healthcare systems. We reported gender distribution of the study populations and performed stratified analyses of outcomes by gender (women, men)

Reporting on race, ethnicity, or other socially relevant groupings

Self-reported race, and ethnicity data in TriNetX comes from the underlying clinical EHR systems of the contributing healthcare systems. We reported race (Black, White, Asian, Unknown race) and ethnicity (Hispanic/Latinx, non-Hispanic/Latinx, unknown ethnicity) distributions of the study populations. We also performed separate analyses of outcomes in study populations stratified by race (Black, White). Other stratified analyses were not performed due to sample size limitation.

Population characteristics

This study does not involve human participants (see below). Population-level characteristics of the study populations are summarized in tables (Table 1-2, Supplementary Table 1-3) in the study.

Recruitment

Not applicable. This is a retrospective cohort study of de-identified and aggregated electronic health records of patients that were accessed from the TriNetX Platform.

Ethics oversight

The TriNetX platform aggregates and HIPAA de-identifies data contributed from the electronic health records of participating healthcare organizations. The TriNetX platform also only reports population-level results (no access to individual patient data) and uses statistical "blurring", reporting all population-level counts between 1 and 10 as 10. Based on the de-identification methods used by TriNetX, as per HIPAA privacy and security rules, TriNetX sought and obtained expert attestation that TriNetX data is HIPAA de-identified (see attached for official expert TriNetX HIPAA deidentification attestation). Because the data in the TriNetX platform is HIPAA de-identified, and therefore, "by definition" is deemed to allow no access to protected health information (and therefore no risk of protected health information disclosure), Institutional Review Boards (IRBs) have no jurisdiction of studies using HIPAA de-identified data. Therefore, IRB approval was neither sought nor obtained for this study.

Note that full information on the approval of the study protocol must also be provided in the manuscript.

Field-specific reporting

Please select the one below that is the best fit for your research. If you are not sure, read the appropriate sections before making your selection.

☒ Life sciences ☐ Behavioural & social sciences ☐ Ecological, evolutionary & environmental sciences

For a reference copy of the document with all sections, see nature.com/documents/nr-reporting-summary-flat.pdf

Life sciences study design

All studies must disclose on these points even when the disclosure is negative.

Sample size	This is a retrospective cohort study with no pre-determined effect size and no sample size calculation was performed. Sample sizes were determined by study populations based on specific inclusion and exclusion cirteria.
Data exclusions	Patients who were prescribed other GLP-1RAs or had bariatric surgery on or before the index event were excluded. This exclusion criteria was pre-established and the goal was to remove the con-founding effects from other GLP1R agonists and from bariatric surgery
Replication	The main study population included patients withobesity who were prescribed semaglutide as Wegovy or non-GLP1R agonists anti-obesity medications during 6/2021-12/2022 and were not prescribed semaglutide prior to 6/2021. Findings were replicated in patients with T2DM who were prescribed semaglutide or non-GLP1R agonists anti-diabetes medications during 12/2017-5/2021. These two study populations are non-overlapping in terms of study periods and exposures
Randomization	Covariates were controlled by propensity-score matching (1:1 using nearest neighbor greedy matching with a caliper of 0.25 times the standard deviation) on covariates
Blinding	We selected cohorts based on the specified inclusion and exclusion criteria. The cohorts were then divided into exposure and comparison cohorts. Participants were aware of the medications that they were prescribed, therefore blinding is not relevant to this study.

Reporting for specific materials, systems and methods

We require information from authors about some types of materials, experimental systems and methods used in many studies. Here, indicate whether each material, system or method listed is relevant to your study. If you are not sure if a list item applies to your research, read the appropriate section before selecting a response.

Materials & experimental systems

n/a	Involved in the study	
☒	☐	Antibodies
☒	☐	Eukaryotic cell lines
☒	☐	Palaeontology and archaeology
☒	☐	Animals and other organisms
☒	☐	Clinical data
☒	☐	Dual use research of concern
☒	☐	Plants

Methods

n/a	Involved in the study	
☒	☐	ChIP-seq
☒	☐	Flow cytometry
☒	☐	MRI-based neuroimaging

Plants

Seed stocks	*Report on the source of all seed stocks or other plant material used. If applicable, state the seed stock centre and catalogue number. If plant specimens were collected from the field, describe the collection location, date and sampling procedures.*
Novel plant genotypes	*Describe the methods by which all novel plant genotypes were produced. This includes those generated by transgenic approaches, gene editing, chemical/radiation-based mutagenesis and hybridization. For transgenic lines, describe the transformation method, the number of independent lines analyzed and the generation upon which experiments were performed. For gene-edited lines, describe the editor used, the endogenous sequence targeted for editing, the targeting guide RNA sequence (if applicable) and how the editor was applied.*
Authentication	*Describe any authentication procedures for each seed stock used or novel genotype generated. Describe any experiments used to assess the effect of a mutation and, where applicable, how potential secondary effects (e.g. second site T-DNA insertions, mosiacism, off-target gene editing) were examined.*
