# Supplementary material for: Associations of semaglutide with incidence and recurrence of alcohol use disorder in real-world population
Source: Nat Commun. 2024 May 28;15:4548. doi: 10.1038/s41467-024-48780-6 (PMC11133479; doi:10.1038/s41467-024-48780-6)

Recurrent AUD diagnosis in patients with obesity and a prior history of AUD during 12−month follow−up time period (comparison between propensity−score matched cohorts)

Population	semaglutide cohort	naltrexone/topiramate cohort	HR (95% CI)
Overall (n = 715/cohort)	21.5% (154)	59.9% (428)	0.25 (0.21−0.30)
Women (n = 291/cohort)	17.5% (51)	54.0% (157)	0.23 (0.17−0.32)
Men (n = 379/cohort)	24.0% (91)	66.2% (251)	0.23 (0.18−0.30)
age <= 55 years (n = 423/cohort)	23.9% (101)	62.6% (265)	0.26 (0.21−0.33)
age > 55 years (n = 260/cohort)	23.1% (60)	53.4% (144)	0.32 (0.23−0.43)
Black (n = 76/cohort)	23.7% (18)	51.3% (39)	0.38 (0.22−0.67)
White (n = 444/cohort)	20.9% (93)	58.6% (260)	0.25 (0.20−0.32)
No T2D (n = 282/cohort)	20.2% (57)	57.4% (162)	0.25 (0.19−0.34)
T2D (n = 134/cohort)	25.4% (34)	61.9% (83)	0.27 (0.18−0.41)

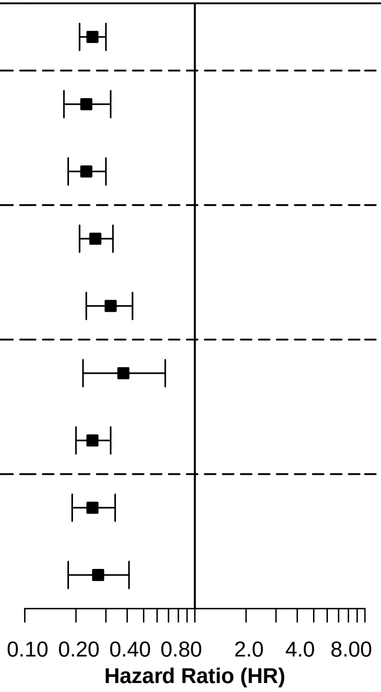

Supplement: Supplementary file 4 — Source Data [file 41467_2024_48780_MOESM4_ESM.zip › semaglutide_AUD/Figure2b.pdf]
