# Supplementary material for: Associations of semaglutide with incidence and recurrence of alcohol use disorder in real-world population
Source: Nat Commun. 2024 May 28;15:4548. doi: 10.1038/s41467-024-48780-6 (PMC11133479; doi:10.1038/s41467-024-48780-6)

Recurrent AUD diagnosis in patients with obesity and a prior history of AUD during 12−month follow−up time period (comparison between propensity−score matched cohorts)

Population	semaglutide cohort	non−GLP−1RA anti−obesity medications cohort	HR (95% CI)
Overall (n = 1,051/cohort)	22.6% (238)	43.0% (452)	0.44 (0.38−0.52)
Women (n = 420/cohort)	19.0% (80)	32.9% (138)	0.51 (0.39−0.67)
Men (n = 553/cohort)	23.9% (132)	46.5% (257)	0.42 (0.34−0.51)
age <= 55 years (n = 586/cohort)	22.9% (134)	43.9% (257)	0.44 (0.35−0.54)
age > 55 years (n = 440/cohort)	23.2% (102)	36.8% (162)	0.55 (0.43−0.70)
Black (n = 140/cohort)	20.7% (29)	37.1% (52)	0.49 (0.31−0.78)
White (n = 699/cohort)	22.7% (159)	41.5% (290)	0.46 (0.38−0.56)
No T2D (n = 540/cohort)	20.6% (111)	41.5% (224)	0.42 (0.33−0.52)
T2D (n = 453/cohort)	24.3% (110)	40.4% (183)	0.50 (0.39−0.63)

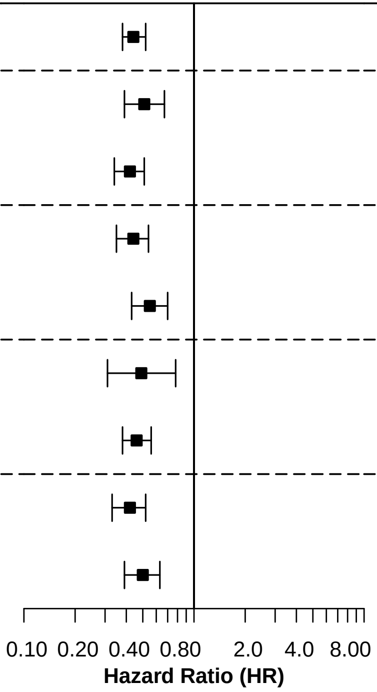

Supplement: Supplementary file 4 — Source Data [file 41467_2024_48780_MOESM4_ESM.zip › semaglutide_AUD/Figure2a.pdf]
