# Supplementary material for: Associations of semaglutide with incidence and recurrence of alcohol use disorder in real-world population
Source: Nat Commun. 2024 May 28;15:4548. doi: 10.1038/s41467-024-48780-6 (PMC11133479; doi:10.1038/s41467-024-48780-6)

Incident AUD diagnosis in patients with T2DM and no prior history of AUD during 12−month follow−up time period (comparison between propensity−score matched cohorts)

Population	semaglutide cohort	non−GLP−1RA anti−diabetes medications cohort	HR (95% CI)
Overall (n = 25,670/cohort)	0.32% (81)	0.52% (134)	0.56 (0.43−0.74)
Women (n = 11,743/cohort)	0.19% (22)	0.34% (40)	0.52 (0.31−0.88)
Men (n = 11,833/cohort)	0.41% (49)	0.73% (86)	0.53 (0.38−0.76)
age <= 55 years (n = 9,974/cohort)	0.34% (34)	0.53% (53)	0.60 (0.39−0.93)
age > 55 years (n = 15,951/cohort)	0.30% (47)	0.53% (84)	0.53 (0.37−0.76)
Black (n = 3,752/cohort)	0.35% (13)	0.51% (19)	0.64 (0.31−1.29)
White (n = 15,452/cohort)	0.28% (43)	0.58% (90)	0.45 (0.31−0.65)
No obesity (n = 10,112/cohort)	0.33% (33)	0.58% (59)	0.51 (0.33−0.78)
Obesity (n = 15,551/cohort)	0.31% (48)	0.47% (73)	0.63 (0.44−0.90)

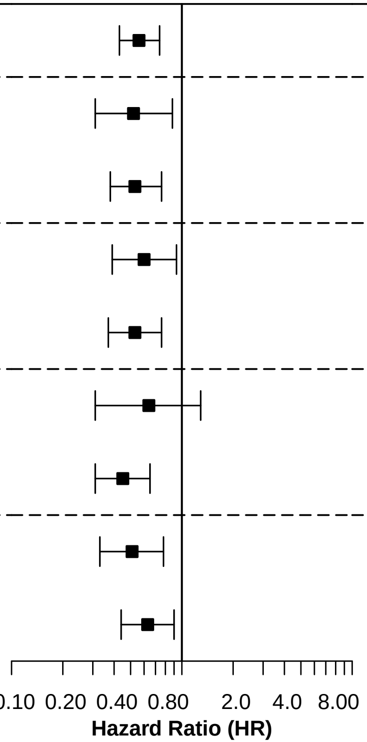

Supplement: Supplementary file 4 — Source Data [file 41467_2024_48780_MOESM4_ESM.zip › semaglutide_AUD/Figure3a.pdf]
