# Supplementary material for: Associations of semaglutide with incidence and recurrence of alcohol use disorder in real-world population
Source: Nat Commun. 2024 May 28;15:4548. doi: 10.1038/s41467-024-48780-6 (PMC11133479; doi:10.1038/s41467-024-48780-6)

Recurrent AUD diagnosis in patients with T2DM and a prior history of AUD during 12−month follow−up time period (comparison between propensity−score matched cohorts)

Population	semaglutide cohort	non−GLP−1RA anti−diabetes medications cohort	HR (95% CI)
Overall (n = 653/cohort)	23.4% (153)	33.2% (217)	0.61 (0.50−0.75)
Women (n = 163/cohort)	20.9% (34)	26.4% (43)	0.73 (0.47−1.15)
Men (n = 443/cohort)	22.1% (98)	31.2% (138)	0.61 (0.47−0.79)
age <= 55 years (n = 258/cohort)	26.7% (69)	37.6% (97)	0.61 (0.45−0.84)
age > 55 years (n = 402/cohort)	20.6% (83)	32.8% (132)	0.55 (0.42−0.72)
Black (n = 104/cohort)	29.8% (31)	34.6% (36)	0.78 (0.49−1.27)
White (n = 370/cohort)	19.7% (73)	29.7% (110)	0.58 (0.43−0.78)
No obesity (n = 195/cohort)	26.2% (51)	41.5% (81)	0.52 (0.37−0.74)
Obesity (n = 463/cohort)	21.2% (98)	33.3% (154)	0.55 (0.43−0.71)

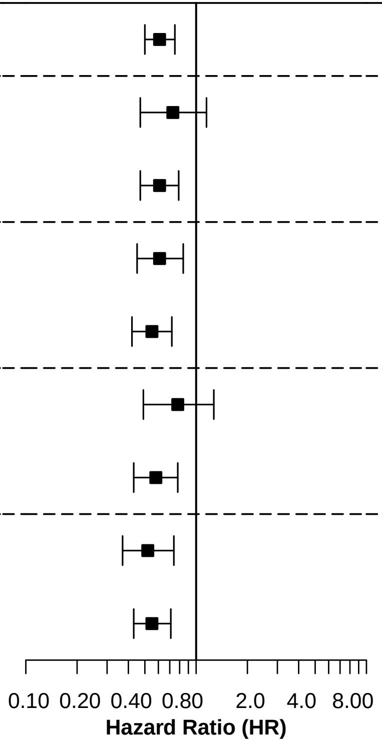

Supplement: Supplementary file 4 — Source Data [file 41467_2024_48780_MOESM4_ESM.zip › semaglutide_AUD/Figure3b.pdf]
