# Supplementary material for: Associations of semaglutide with incidence and recurrence of alcohol use disorder in real-world population
Source: Nat Commun. 2024 May 28;15:4548. doi: 10.1038/s41467-024-48780-6 (PMC11133479; doi:10.1038/s41467-024-48780-6)

Incident and recurrent AUD diagnosis in patients with T2DM at longer follow−up time period (comparison between propensity−score matched cohorts)

Follow−up	semaglutide cohort	non−GLP−1RA anti−diabetes medications cohort	HR (95% CI)
**Incident AUD (n=25,670/cohort)**			
1−year	0.32% (81)	0.52% (134)	0.56 (0.43−0.74)
2−year	0.58% (149)	0.90% (231)	0.59 (0.48−0.73)
3−year	0.90% (232)	1.19% (305)	0.72 (0.60−0.85)
**Recurrent AUD (n=653/cohort)**			
1−year	23.4% (153)	33.2% (217)	0.61 (0.50−0.75)
2−year	29.3% (191)	38.9% (294)	0.64 (0.53−0.77)
3−year	33.5% (219)	41.7% (272)	0.68 (0.57−0.81)

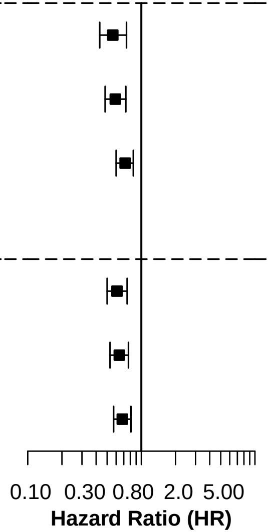

Supplement: Supplementary file 4 — Source Data [file 41467_2024_48780_MOESM4_ESM.zip › semaglutide_AUD/Figure3c.pdf]
