# Supplementary material for: Associations of semaglutide with incidence and recurrence of alcohol use disorder in real-world population
Source: Nat Commun. 2024 May 28;15:4548. doi: 10.1038/s41467-024-48780-6 (PMC11133479; doi:10.1038/s41467-024-48780-6)

Incident AUD diagnosis in patients with obesity and no prior history of AUD during 12−month follow−up time period (comparison between propensity−score matched cohorts)

Population	semaglutide cohort	non−GLP−1RA anti−obesity medications cohort	HR (95% CI)
Overall (n = 26,566/cohort)	0.37% (98)	0.73% (193)	0.50 (0.39−0.63)
Women (n = 17,977/cohort)	0.22% (40)	0.44% (79)	0.50 (0.34−0.73)
Men (n = 6,903/cohort)	0.59% (41)	1.14% (79)	0.50 (0.35−0.74)
age <= 55 years (n = 15,767/cohort)	0.30% (48)	0.61% (96)	0.49 (0.35−0.70)
age > 55 years (n = 10,440/cohort)	0.48% (50)	0.86% (90)	0.54 (0.38−0.76)
Black (n = 4,107/cohort)	0.32% (13)	0.71% (29)	0.43 (0.23−0.83)
White (n = 17,861/cohort)	0.35% (62)	0.67% (120)	0.51 (0.38−0.69)
No T2DM (n = 17,609/cohort)	0.39% (68)	0.60% (106)	0.64 (0.47−0.87)
T2DM (n = 8,696/cohort)	0.30% (26)	0.90% (78)	0.32 (0.20−0.49)

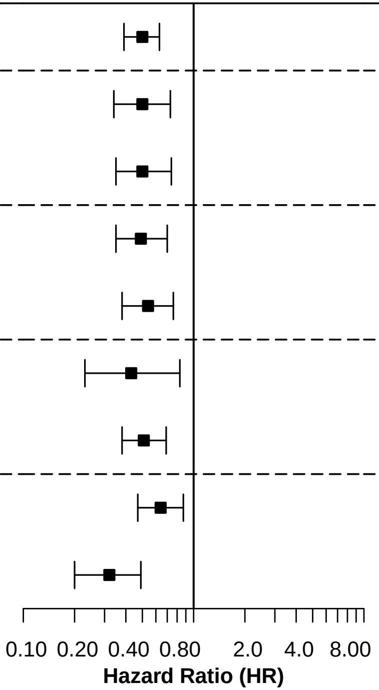

Supplement: Supplementary file 4 — Source Data [file 41467_2024_48780_MOESM4_ESM.zip › semaglutide_AUD/Figure1a.pdf]
