# Supplementary material for: Associations of semaglutide with incidence and recurrence of alcohol use disorder in real-world population
Source: Nat Commun. 2024 May 28;15:4548. doi: 10.1038/s41467-024-48780-6 (PMC11133479; doi:10.1038/s41467-024-48780-6)

Incident AUD diagnosis in patients with obesity and no prior history of AUD during 12−month follow−up time period (comparison between propensity−score matched cohorts)

Population	semaglutide cohort	naltrexone/topiramate cohort	HR (95% CI)
Overall (n = 15,097/cohort)	0.35% (53)	0.78% (118)	0.44 (0.32−0.61)
Women (n = 10,718/cohort)	0.24% (26)	0.61% (65)	0.39 (0.25−0.62)
Men (n = 3,315/cohort)	0.57% (19)	1.36% (45)	0.41 (0.24−0.70)
age <= 55 years (n = 9,642/cohort)	0.32% (31)	0.72% (69)	0.44 (0.29−0.68)
age > 55 years (n = 5,289/cohort)	0.45% (24)	0.83% (44)	0.53 (0.32−0.87)
Black (n = 2,511/cohort)	<0.40% (<10)	0.80% (20)	0.24 (0.09−0.65)
White (n = 9,808/cohort)	0.38% (37)	0.79% (77)	0.48 (0.32−0.71)
No T2DM (n = 11,335/cohort)	0.40% (45)	0.72% (81)	0.56 (0.39−0.80)
T2DM (n = 3,610/cohort)	<0.28% (<10)	1.00% (36)	0.26 (0.13−0.53)

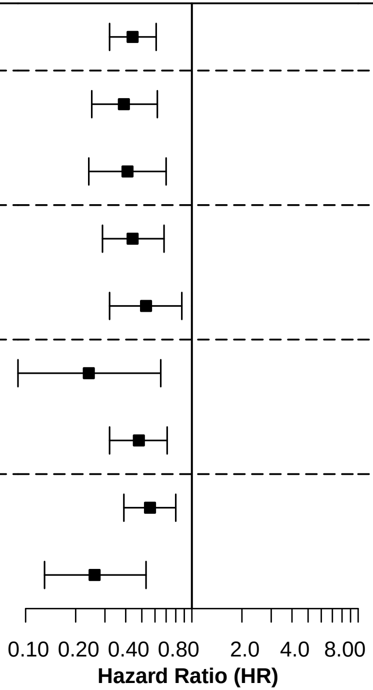

Supplement: Supplementary file 4 — Source Data [file 41467_2024_48780_MOESM4_ESM.zip › semaglutide_AUD/Figure1b.pdf]
